# Successful treatment of intubation-induced severe neurogenic post-extubation dysphagia using pharyngeal electrical stimulation in a COVID-19 survivor: a case report

**DOI:** 10.1186/s13256-021-02763-z

**Published:** 2021-03-22

**Authors:** Marianna Traugott, Wolfgang Hoepler, Reinhard Kitzberger, Sophie Pavlata, Tamara Seitz, Sebastian Baumgartner, Gudrun Placher-Sorko, Daniela Pirker-Krassnig, Urs Ehehalt, Andreas Grasnek, Michaela Beham-Kacerovsky, Emanuela Friese, Christoph Wenisch, Stephanie Neuhold

**Affiliations:** 1 Fourth Medical Department with Infectious Diseases and Tropical Medicine, Klinik Favoriten – Kaiser Franz Josef Hospital, Kundratstraße 3, 1100 Vienna, Austria; 2 Otorhinolaryngeology Department, Klinik Favoriten – Kaiser Franz Josef Hospital, Kundratstraße 3, 1100 Vienna, Austria

**Keywords:** COVID-19 infection, Post-extubation dysphagia (PED), Pharyngeal electrical stimulation (PES), Intensive care unit (ICU), Case report

## Abstract

**Background:**

A significant portion of critically ill patients with coronavirus disease 2019 (COVID-19) are at high risk of developing intensive care unit (ICU)-acquired swallowing dysfunction (neurogenic dysphagia) as a consequence of requiring prolonged mechanical ventilation. Pharyngeal electrical stimulation (PES) is a simple and safe treatment for neurogenic dysphagia. It has been shown that PES can restore safe swallowing in orally intubated or tracheotomized ICU patients with neurogenic dysphagia following severe stroke. We report the case of a patient with severe neurogenic post-extubation dysphagia (PED) due to prolonged intubation and severe general muscle weakness related to COVID-19, which was successfully treated using PES.

**Case presentation:**

A 71-year-old Caucasian female patient with confirmed severe acute respiratory syndrome coronavirus 2 (SARS-CoV-2) infection developed neurogenic dysphagia following prolonged intubation in the ICU. To avoid aerosol-generating procedures, her swallowing function was evaluated non-instrumentally as recommended by recently published international guidelines in response to the COVID-19 pandemic. Her swallowing function was markedly impaired and PES therapy was recommended. PES led to a rapid improvement of the PED, as evaluated by bedside swallowing assessments using the Gugging Swallowing Screen (GUSS) and Dysphagia Severity Rating Scale (DSRS), and diet screening using the Functional Oral Intake Scale (FOIS). The improved swallowing, as reflected by these measures, allowed this patient to transfer from the ICU to a non-intensive medical department 5 days after completing PES treatment.

**Conclusions:**

PES treatment contributed to the restoration of a safe swallowing function in this critically ill patient with COVID-19 and ICU-acquired swallowing dysfunction. Early clinical bedside swallowing assessment and dysphagia intervention in COVID-19 patients is crucial to optimize their full recovery. PES may contribute to a safe and earlier ICU discharge of patients with ICU-acquired swallowing dysfunction. Earlier ICU discharge and reduced rates of re-intubation following PES can help alleviate some of the pressure on ICU bed capacity, which is critical in times of a health emergency such as the ongoing COVID-19 pandemic.

## Background

Following the global spread of the novel severe acute respiratory syndrome coronavirus 2 (SARS-CoV-2) infection, which first emerged in China in December 2019, the World Health Organization (WHO) declared the coronavirus disease 2019 (COVID-19) outbreak a pandemic on March 11, 2020. The percentages (9–32%) of severely or critically ill COVID-19 patients admitted to intensive care units (ICU) differed among the affected countries [1–4]. Overall, 20–40% of patients in the United States required mechanical ventilation by the time of their ICU admission [5].

Prolonged ICU stay—especially a prolonged duration of mechanical ventilation coupled with sedation and bed rest—is associated with neurological disorders due to a reduction in afferent sensitivity; consequently, there is a high risk of dysfunction of the swallowing musculature, which can persist for months or years after ICU discharge [6]. COVID-19 can also manifest with neurological symptoms [7], both myopathy and myositis [8], which aggravate post-extubation dysphagia (PED). Indeed, the trauma caused by the endotracheal tube is considered one of the main causes of ICU-acquired dysphagia [9]. PED is defined as swallowing difficulty after extubation. In a systematic review including nine clinical studies analyzing 775 ICU patients after oral endotracheal intubation, 49% of patients presented with PED [9]. A large prospective observational trial (DYnAMICS) evaluating dysphagia in 1304 mechanically ventilated, unselected ICU patients reported PED incidence of 18.3%. Moreover, in that study, among the 90 patients discharged from the hospital, 58 (64.4%) showed persistent swallowing dysfunction [10]. Dysphagia complications following prolonged hospitalization are well known and are associated with a high risk of malnutrition and dehydration, decreased health-related quality of life, and increased occurrence of aspiration pneumonia.

**Fig. 1:**
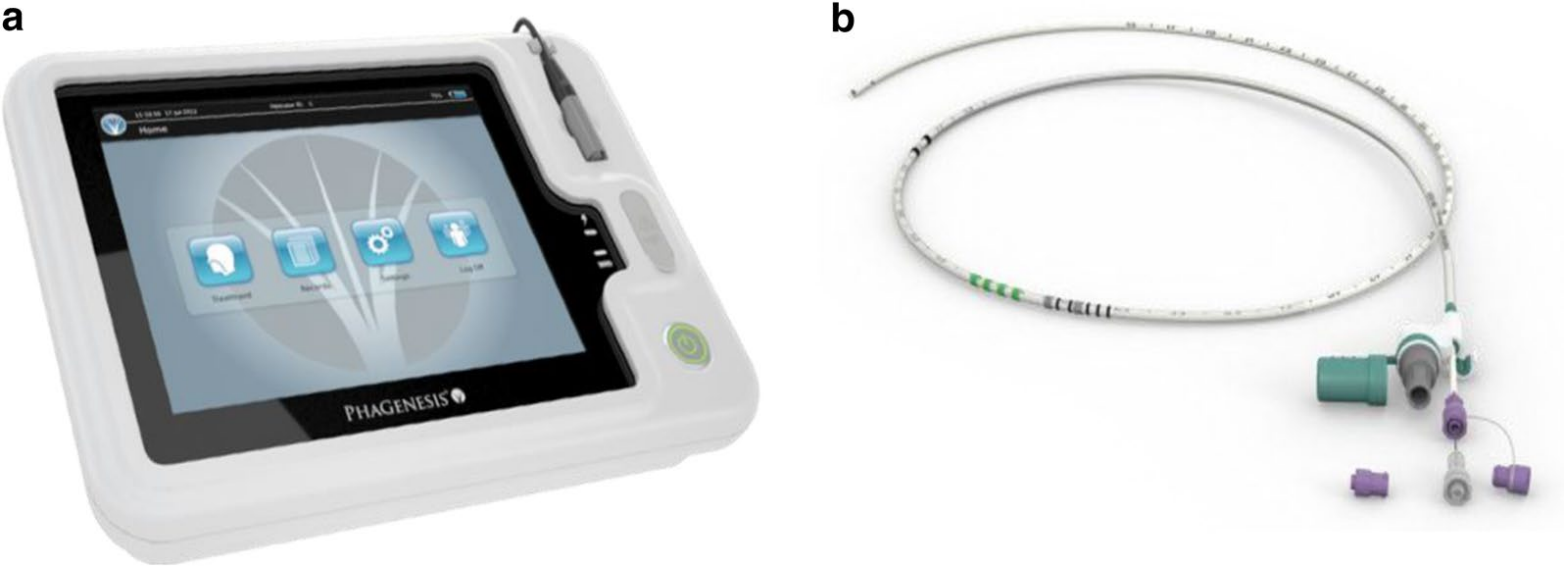
Description of Phagenyx^®^ system, a medical device comprising a base station with a touch screen user interface (**a**) and a sterile single-patient-use catheter (**b**) that can be used to deliver nutrition and hydration for up to 30 days after insertion

Currently, three different therapeutic options exist for treatment of oropharyngeal dysphagia: (i) patient-specific variation in dietary texture, (ii) postural changes/compensatory maneuvers, and (iii) medical devices delivering personalized neuromuscular stimulation to improve swallowing function by treating the afferent sensory pathways involved in the pharyngeal impairment [13]. The Phagenyx^®^ system (a medical device CE-marked since 2012 from Phagenesis Ltd., Manchester, UK) was designed to treat the symptoms of swallowing disorders by stimulating sensory nerves in the oropharynx through directed electrical pulses. Pharyngeal electrical stimulation (PES) uses a nasogastric feeding tube-like stimulation catheter incorporating two specially designed electrodes (Fig. 1). Through communication with the patient, the healthcare worker finds an individually adjusted stimulation intensity for optimized PES. Typically, stimulation is delivered using a current intensity range between 1 and 50 mA, a stimulation frequency of 5 Hz, and a pulse width of 200 μs. Each period of PES usually lasts 10 minutes. Three treatment sessions may be given on several consecutive days to patients with neurogenic dysphagia [14, 15]. PES therapy has been used to safely and successfully treat neurogenic dysphagia in patients presenting with multiple medical conditions: in orally intubated ICU patients [15], in tracheotomized severely dysphagic stroke patients [14, 16, 17], in patients with brain injury [18], and patients suffering from multiple sclerosis [19]. Moreover, a large European registry (PHADER registry) in which over 230 patients with neurogenic dysphagia were successfully treated with Phagenyx^®^ was recently reported [20]. Despite its frequent occurrence in intubated patients and significant morbidity, there is a lack of awareness of the need to systematically screen patients for PED and its consequences, to allow earlier intervention and treatment [10].

We describe the case of a COVID-19 patient presenting with severe general muscle weakness and ICU-acquired swallowing dysfunction who—despite her viral infection and prolonged ICU stay—was successfully treated with PES (post-extubation). This enhanced safe swallowing allowed the patient to resume a more normal nutritional intake, avoided re-intubation or tracheostomy, and therefore permitted a stable ICU discharge.

## Case presentation

### Patient’s relevant demographic details and medical history

**Fig. 2:**
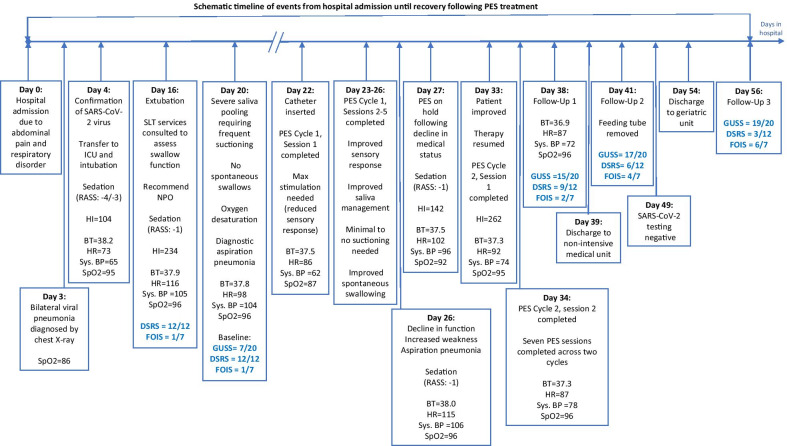
Schematic timeline of events from hospital admission until recovery after PES treatment. *BT* body temperature (°C), *HI* Horowitz oxygenation index, *HR* heart rate (beats per minute), *BP* blood pressure (mean, mmHg), *SpO*_*2*_ oxygen saturation (%)

The 71-year-old Caucasian female patient had a medical history significant for bilateral hydronephrosis, bilateral sub-pelvic ureteral obstruction, pneumococcal pneumonia (2008), and restless leg syndrome. She presented to the urology department of a partnering public hospital in Vienna (Austria) on April 3, 2020, with complaints of abdominal pain and a dry cough (see Fig. 2 for a schematized summary of patient’s hospitalization timeline). On day 3 after admission, a chest X-ray was performed, which showed a typical bilateral viral pneumonia. Upon initial assessment, the patient demonstrated partial respiratory insufficiency, with peripheral oxygen saturation (SpO_2_) of 86%. On day 4, the respiratory insufficiency deteriorated quickly (10 L/minute oxygen required to maintain SpO_2_>90%), so the patient was transferred to the intensive care unit (ICU) and intubated. At that time, her Horowitz index (HI) reached 104, corresponding to a moderately severe lung injury [21, 22]. Laboratory tests revealed lymphopenia and elevated C-reactive protein (CRP), both typical biomarkers of COVID-19 [1, 2]. After confirmation of SARS-CoV-2 through polymerase chain reaction (PCR) on a nasopharyngeal swab, the patient was transferred (mechanically ventilated) to our ICU, being the main ICU in charge of managing COVID-19 patients in Vienna (IV Med. Dept., Kaiser Franz Josef Hospital). Prior to hospitalization, the patient was living independently and was safely managing a regular solid and thin liquid diet with no history of dysphagia.

### Antiviral and antibiotic therapies

Hydroxychloroquine was initiated on day 7 after admission as an antiviral treatment for COVID-19 and discontinued on day 8 due to prolongation of the corrected Q-T interval (QTc). Subsequently, the patient received standard supportive care for a viral pneumonia. Following the initial diagnostic PCR on day 4, PCR for SARS-CoV-2 was performed on tracheal secretions and nasopharyngeal swabs twice a week (as long as secretions could be aspirated). On day 21, the PCR of the nasopharyngeal swab was only weakly positive, while tracheal secretion was still clearly positive. On day 49, the nasopharyngeal swab sample was negative for the first time, at that time tracheal secretion could not be obtained for testing.

On initial admission, ampicillin/sulbactam was started empirically. After transfer to the COVID-19 ICU, the antibacterial treatment was switched to piperacillin/tazobactam, which was stopped after 7 days of treatment in the absence of evidence of a bacterial infection. On day 22, increased putrid pulmonary secretions were observed, and a PCR test was conducted on the patient’s sputum (pneumonia panel plus, bioMérieux^®^) targeting 33 different kinds of bacteria and viruses. *Klebsiella pneumoniae* was detected and antimicrobial therapy was started with cefepime. The patient’s general condition worsened on day 27 (see vital signs indicated in Fig. 2); as she also developed elevated inflammation parameters (leukocytes and CRP), the antibiotic therapy was escalated to meropenem. The patient’s condition then improved (see vital signs on day 33 indicated in Fig. 2), and the therapy was de-escalated to ceftazidime, which was further administered between days 32 and 36.

### Respiratory support

From day 4 until day 16 of the hospital stay, the patient was mechanically ventilated via endotracheal tube for 12 days and dependent on catecholamine support. Following extubation, the patient received noninvasive ventilation (NIV) with a full face mask (HI=234, indicating a mild lung injury). The next day, she was transitioned to high-flow nasal cannula (HFNC). However, on day 25, the patient’s general health condition deteriorated rapidly (HI=142, severe lung injury). Hence, continuous NIV with full face mask was necessary again until day 29, after which high-flow nasal cannula was restarted. On day 35, the patient was finally put on oxygen insufflation through low-flow nasal cannula and weaned to breathing room air in the following days (HI=262, mild lung injury).

### Baseline dysphagia assessments

Speech and language therapy services were consulted following endotracheal extubation on day 16 to assess the swallowing function. These assessments were performed initially at bedside; they revealed oral and suspected pharyngeal swallowing dysfunction manifest as poor saliva management with observed drooling, suspected reduced hyolaryngeal elevation as judged via palpation during volitional swallowing, and aphonia. Consequently, “nil by mouth” was recommended by the speech and language therapist (SLT) (Dysphagia Severity Rating Scale score [23], DSRS = 12; Functional Oral Intake Scale score [24], FOIS = 1). Due to the COVID-19 pandemic and restrictions on the conduct of aerosol-generating procedures, the SLT was unable to complete an instrumental assessment of the swallow, such as a fibreoptic endoscopic evaluation of swallowing (FEES) or videofluoroscopy (VFS), to more directly assess the patient’s oropharyngeal swallowing function. Hence, dysphagia evaluations were limited to clinical bedside screening and noninvasive assessment tools.

**Fig. 3:**
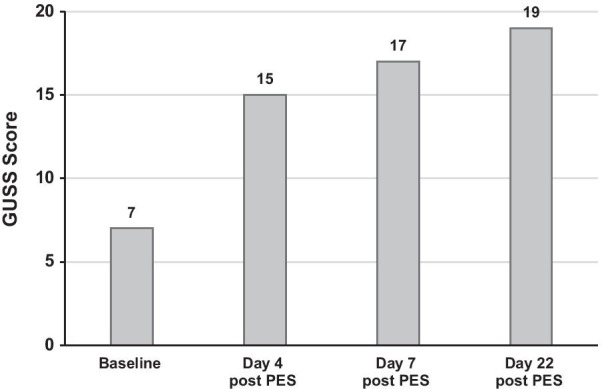
Gugging Swallowing Screen (GUSS) dysphagia assessments. A 20-point GUSS scale determining the dysphagia severity and the risk of aspiration (20 being the best score: no dysphagia and minimal aspiration risk) was used at baseline, day 4, day 7, and day 22 following the final PES treatment session

**Fig. 4:**
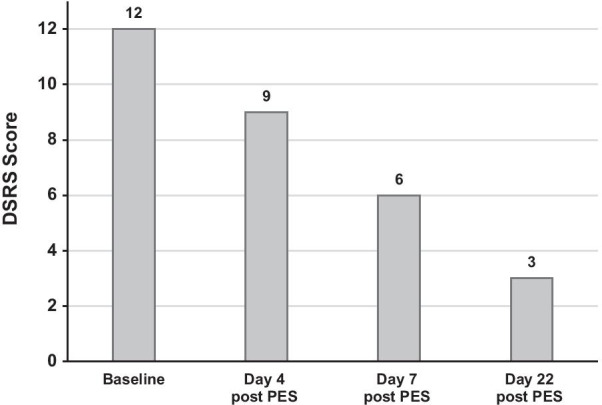
Dysphagia Severity Rating Scale (DSRS) dysphagia assessments. A 12-point DSRS scale grading dysphagia severity based on fluid and diet modification, as well as supervision requirements for feeding (12 being the worst score: no oral fluids or feeding, even under supervision), was used at baseline, day 4, day 7, and day 22 following the final PES treatment session

**Fig. 5:**
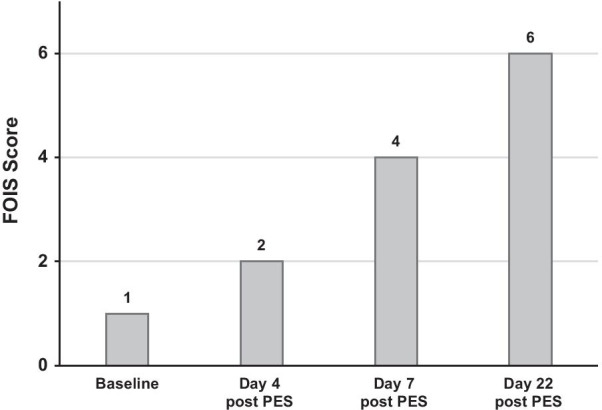
Functional Oral Intake Scale (FOIS) dysphagia assessments. A 7-point FOIS scale reflecting food and liquids intake by mouth on a consistent basis (7 being the best score: normal oral intake without any restrictions) was used at baseline, day 4, day 7, and day 22 following the final PES treatment session

On day 18, the SLT performed a clinical bedside swallow evaluation using the Gugging Swallowing Screen (GUSS) [19]; the patient reached a total score of 6 out of 20, indicating the presence of “severe dysphagia with a high risk of aspiration.” The diet recommendation of nil by mouth (DSRS = 12; FOIS = 1) was maintained. During the screening, the patient continued to demonstrate poor management of oropharyngeal secretions, with minimal reflexive swallowing, and required frequent suctioning. The SLT administered a trial of thickened water, which was followed by an unproductive cough response, further impacted by continued generalized muscle weakness, a typical feature seen in COVID-19 patients. On day 20, the swallow function was re-assessed via GUSS and the patient received a score of 7 out of 20 (Fig. 3), again indicating “severe dysphagia with a high risk of aspiration,” with continued recommendation of nil by mouth [DSRS = 12 (Fig. 4); FOIS= 1 (Fig. 5)]; these represented the initial GUSS, DSRS, and FOIS baseline values prior to starting PES on day 22. Informally, the patient was observed having increased difficulty managing secretions, required more frequent suctioning, and had an oxygen saturation of 86%. Subsequently, on day 20 and day 26, the patient developed aspiration pneumonia requiring antibiotic treatment.

### PES treatment

Due to persistent severe dysphagia, two cycles of PES therapy were performed: a first cycle between days 22 and 26 (five sessions) and a second cycle on days 33 and 34 (two sessions). Each daily session lasted 10 minutes. This patient was the first COVID-19 patient receiving PES in our department. The delayed start of PES therapy was due to unfounded ambiguities within the team concerning hygiene matters. Stimulation parameters (current, mA) are described in Table 1.

**Table 1:** Pharyngeal electrical stimulation (PES) current intensities

	PES cycle 1					PES cycle 2	
Date of PES	Day 22	Day 23	Day 24	Day 25	Day 26	Day 33	Day 34
Stimulation (mA)	48	15	22	19	13	16	12

Following the first three sessions of PES treatment, swallowing improvements were observed, including improved secretion management and increased reflexive swallowing; however, the patient’s medical condition deteriorated on day 25, with increasing inflammation parameters. The persistent extreme muscle weakness caused a moribund state. Hence, on day 27, the behavioral therapy, including PES, was paused. Because of stress related to the necessary NIV treatment, the patient was sedated until day 29, when clinical improvement was observed (normal heart and breathing rates, good vigilance). On day 32, the patient’s medical status and vigilance were stable enough to restart standard behavioral treatment. Therefore, a second PES cycle was performed on days 33 and 34, with the patient receiving seven stimulation sessions in total across two treatment cycles (Table 1).

### Treatment outcome

On day 38, 4 days following the final PES treatment session, the SLT re-evaluated swallowing and noted a GUSS total score of 15 out of 20, describing “slight dysphagia with a low risk of aspiration”—a significant improvement from baseline GUSS scoring of 7 out of 20 (Fig. 3). In light of these functional improvements, the patient’s diet was advanced from nil by mouth (DSRS = 12; FOIS = 1) to mushy homogeneous solid food (International Dysphagia Diet Standardisation Initiative [25], IDDSI = 4) and thickened fluids (IDDSI = 3) administered in teaspoon amounts using a compensatory chin tuck posture for increased swallow safety (DSRS = 9; FOIS = 2) (Figs. 4 and 5). On day 41, GUSS was re-assessed, with additional improvement noted, as the patient scored 17 out of 20, indicating “slight dysphagia with a low risk of aspiration” (Fig. 3); the patient’s diet was advanced to semisolids with soft-consistency foods (for example white bread, IDDSI = 5) and concentrated fluid (IDDSI = 2), given while the patient was in an upright position in a transverse bed during meals (DSRS = 6; FOIS = 4) (Figs. 4 and 5). It is important to note that due to the patient’s overall improved swallowing function and safety, she was discharged to a non-intensive medical department on day 39, and the feeding tube was removed on day 41. Final post-treatment GUSS was completed on day 56 and revealed a total score of 19/20 (Fig. 3). The patient’s diet was upgraded to soft solid foods (IDDSI = 6) and thin liquids (IDDSI = 0) (DSRS = 3; FOIS = 6) (Figs. 4 and 5).

On May 27, 2020 (day 54), the patient was transferred to acute geriatrics for further intensive physiotherapeutic mobilization, with the objective of returning to living independently at home again.

## Discussion and conclusions

We are presenting the first case report of a COVID-19 patient with ICU-acquired PED, showing full recovery of swallowing function following PES therapy, and importantly, in the absence of any PES-related adverse events. COVID-19-associated symptoms need to be taken into consideration in the treatment of dysphagic patients, as these patients seem to be at increased risk for healthcare-associated infections. Indeed, bacterial co-infections, as observed in this patient, seem to be a typical characteristic of severe and critical forms of SARS-CoV-2 infection [26], along with severe muscle weakness [27].

PED, which has been reported by up to 62% of patients with severe COVID-19 requiring mechanical ventilation, is one of the mid- to long-term sequelae [11]. Critically ill COVID-19 dysphagic patients seem to be particularly at higher risk of aspiration and subsequent aspiration pneumonia; indeed, brain areas and peripheral nerves and muscles, which are responsible for normal deglutition, are often impaired as a consequence of COVID-19 [12, 28]. The swallowing dysfunction observed in the previously dysphagia-naïve patient reported here is most likely the result of endotracheal tube trauma, a commonly observed problem in cases of intubation in an emergency setting [10]. Our observations, as reported in a recent publication [29], also suggest that there is a direct neurological component involved in the pathology of COVID-19 patients. Further research will nevertheless be needed to understand the full extent of this novel coronavirus disease and its consequences.

Of note, a high stimulation level was required on the first day of PES, showing the lack of neuromuscular sensitivity by this patient following her prolonged (12-day) intubation; such reduced local sensitivity has been previously described in patients with ICU-acquired dysphagia [12]. From the second stimulation day, a more normal range of stimulation values were used according to patient responses, showing a recovery of sensibility after a single 10-minute PES treatment session. PES can be delivered while patients are still intubated as well; in this case, the stimulation threshold can be deduced from more autonomic cues from lightly sedated patients (Richmond Agitation-Sedation Scale [RASS]-1), such as sweating, mimics, increased blood pressure, or stress. A recent pilot study by Koestenberger *et al.* showed a significantly earlier improvement in swallowing after PES treatment in orally intubated ICU patients compared to those stimulated following extubation, suggesting a faster recovery of dysphagia when PES is performed sooner [15]. Moreover, patients receiving PES treatment had a lower prevalence of pneumonia and frequency of re-intubation than patients without PES stimulation. Had we started PES therapy during intubation in this patient, we may have accelerated her recovery by minimizing the consequences of dysphagia.

During our patient’s ICU stay, the ongoing therapeutic measures had to be postponed—including PES dysphagia therapy for 6 days—due to the *de novo* sedation of the patient because of the deterioration of her general medical state; however, thanks to the first cycle of PES, a re-intubation or tracheostomy was avoided.

At the beginning of the COVID-19 outbreak, the priority for ICUs was simply to keep patients alive, and therefore no therapeutic procedures other than those associated with intensive care were performed. As time progressed, medical staff gained experience and came slowly back to normality. A balance had to be found between the limited exposure of medical staff and the rights of critically ill COVID-19 ICU patients for an optimal health-related quality of life and the full program of medical care they needed. In this sense, a recently published WHO guideline recommends continuous rehabilitation interventions for patients with severe COVID-19 [30].

For ICU patients, systematic bedside screening for PED—for which a feasible and pragmatic approach has already been published [12]—as well as rehabilitation measures of neurogenic dysphagia should be implemented into clinical protocols. To minimize the risk of viral exposure through aerosol emissions during dysphagia therapy, medical staff should wear adequate personal protective equipment (PPE) for procedures on suspected or confirmed COVID-19 cases [31, 32]. This will allow early dysphagia assessment and the subsequent implementation of personalized treatment [12]. COVID-19 survivors in the acute, subacute, and long-term phases of care have specific rehabilitation needs; PES therapy can be used to facilitate early recovery, further transfer of dysphagic patients to non-intensive case rehabilitation facilities, and contribute overall to shorten hospital length of stay.

Clinical management guidelines related to dysphagia therapy, as well as international intensive care societies such as the ÖGARI [Österreichische Gesellschaft für Anästhesiologie, Reanimation und Intensivmedizin] in Austria, recommended in their recently published position statements that aerosol-generating procedures be restricted on COVID-19-positive or suspected patients in order to avoid contamination risk for health care personal [29, 33]. Consequently, only non-instrumental bedside examinations are performed to assess dysphagia in potentially infected ICU patients [26–28]. Although instrumental assessments in COVID-19-positive cases are currently limited, optimal rehabilitation of PED following prolonged ICU stay is feasible; the use of SLT interventions or equipment-based therapies like PES leads to effective and early management of dysphagia [34]. A holistic approach within an intensive care multidisciplinary team is needed to optimally take care of patients with acute and early post-acute neurogenic dysphagia. In the absence of any established standard treatments for dysphagic COVID-19 patients, PES may facilitate early discharge to non-intensive care units and reduce the risk of ICU readmission. As we have unfortunately learned from the ongoing COVID-19 pandemic, maintaining adequate ICU efficiency and bed capacity is a critical factor in preventing the health care system from being overwhelmed.

## Data Availability

Not applicable.

## Abbreviations

BT: Body temperature
DSRS: Dysphagia Severity Rating Scale
FEES: Fibreoptic Endoscopic Evaluation of Swallowing
FOIS: Functional Oral Intake Scale
GUSS: Gugging Swallowing Screen
HFNC: High-flow nasal cannula
HI: Horowitz oxygenation index
HR: Heart rate
IDDSI: International Dysphagia Diet Standardisation Initiative
NIV: Noninvasive ventilation
PED: Post-extubation dysphagia
PES: Pharyngeal electrical stimulation
RASS: Richmond Agitation-Sedation Scale
BP: Blood pressure
SpO_2_: Oxygen saturation
VFS: Videofluoroscopy
